# Host–microbiome interactions in breast cancer progression and treatment response

**DOI:** 10.3389/fmed.2026.1827694

**Published:** 2026-05-25

**Authors:** Jhommara Bautista, Maria Elena Lara-Hernández, Michelle Hidalgo-De La Cruz, Verónica Andino-Araque, Mauricio León-Rivera, Andrés López-Cortés

**Affiliations:** 1 Cancer Research Group (CRG), Faculty of Medicine, Universidad de Las Américas, Quito, Ecuador; 2 Hospital Metropolitano, Quito, Ecuador; 3 Hospital Axxis, Quito, Ecuador; 4 Clínica Ricardo Palma, San Isidro, Peru

**Keywords:** bioactive compounds, breast cancer, endocrine pathways, inflammatory signaling, metabolic regulation

## Abstract

Breast cancer (BC) is a biologically heterogeneous disease in which tumor progression and therapeutic response vary substantially across patients and molecular subtypes. Alongside genetic, endocrine, and immunological determinants, microbial ecosystems have been proposed as components of the host environment that interact with tumor biology. Microorganisms detected in breast tissue, the gastrointestinal tract, and the oral cavity coexist with epithelial and immune compartments and participate in metabolic and inflammatory processes relevant to mammary physiology. Differences in microbial composition have been reported between non-malignant and malignant breast tissue, while intestinal microbial metabolism generates bioactive compounds capable of interacting with immune regulation and systemic endocrine signaling. Microbial enzymatic activity involved in estrogen deconjugation further connects intestinal ecology with hormone-responsive disease. Microbiome-related variation has also been examined in relation to systemic therapies, where differences in microbial composition have been observed alongside variability in therapeutic outcomes. This review examines current knowledge on host-microbiome interactions across breast, gut, and oral environments and discusses how microbial ecology intersects with inflammatory signaling, metabolic regulation, and endocrine pathways relevant to breast cancer progression and treatment response. Methodological challenges and future research directions for microbiome-informed oncology are also considered.

## Introduction

Breast cancer (BC) remains the most frequently diagnosed malignancy among women worldwide and one of the leading causes of cancer-related mortality. According to GLOBOCAN 2022, approximately 2.3 million new cases and between 666,000 and 685,000 deaths were reported globally, representing nearly 24% of all female cancers and about 15% of cancer deaths among women. Incidence rates are highest in economically developed regions, although the absolute number of cases is greatest in East Asia due to population size. Global projections indicate that by 2050 the burden of BC may increase substantially, with estimated rises exceeding 50% in incidence and more than 70% in mortality if current trends persist (1, 2). Despite improvements in screening programs and therapeutic strategies, considerable variability in disease progression and treatment response continues to be observed across molecular subtypes and patient populations. This heterogeneity reflects the complex biological environment in which breast tumors develop, involving interactions among genetic susceptibility, endocrine signaling, immune regulation, environmental exposures, and metabolic processes (3).

The human microbiome has emerged as a component of the host biological environment associated with cancer. Microbial communities inhabit multiple anatomical sites and form ecological systems that coexist with epithelial, stromal, and immune compartments. The detection of microbial DNA and viable microorganisms in mammary tissue has challenged the concept of sterility in internal organs. Breast tissue has been reported to harbor bacterial communities dominated by Proteobacteria, Firmicutes, and Actinobacteria, with compositional differences observed between non-malignant and tumor samples (4, 5).

Microbial communities located outside the breast may also be relevant in this context. The intestinal and oral microbiomes represent major reservoirs of microorganisms within the human body, and differences in microbial composition have been described across these compartments in individuals with BC. Evidence suggests that microbial ecosystems at distinct anatomical sites may be interconnected, although the extent and functional relevance of this relationship remain under investigation (6, 7).

Current research has explored associations between microbial profiles and clinical outcomes in BC, including disease progression and response to therapy. Differences in microbial composition have been reported across disease stages and treatment settings, suggesting that microbial dynamics may change throughout the clinical course of BC (8, 9).

**FIGURE 1:**
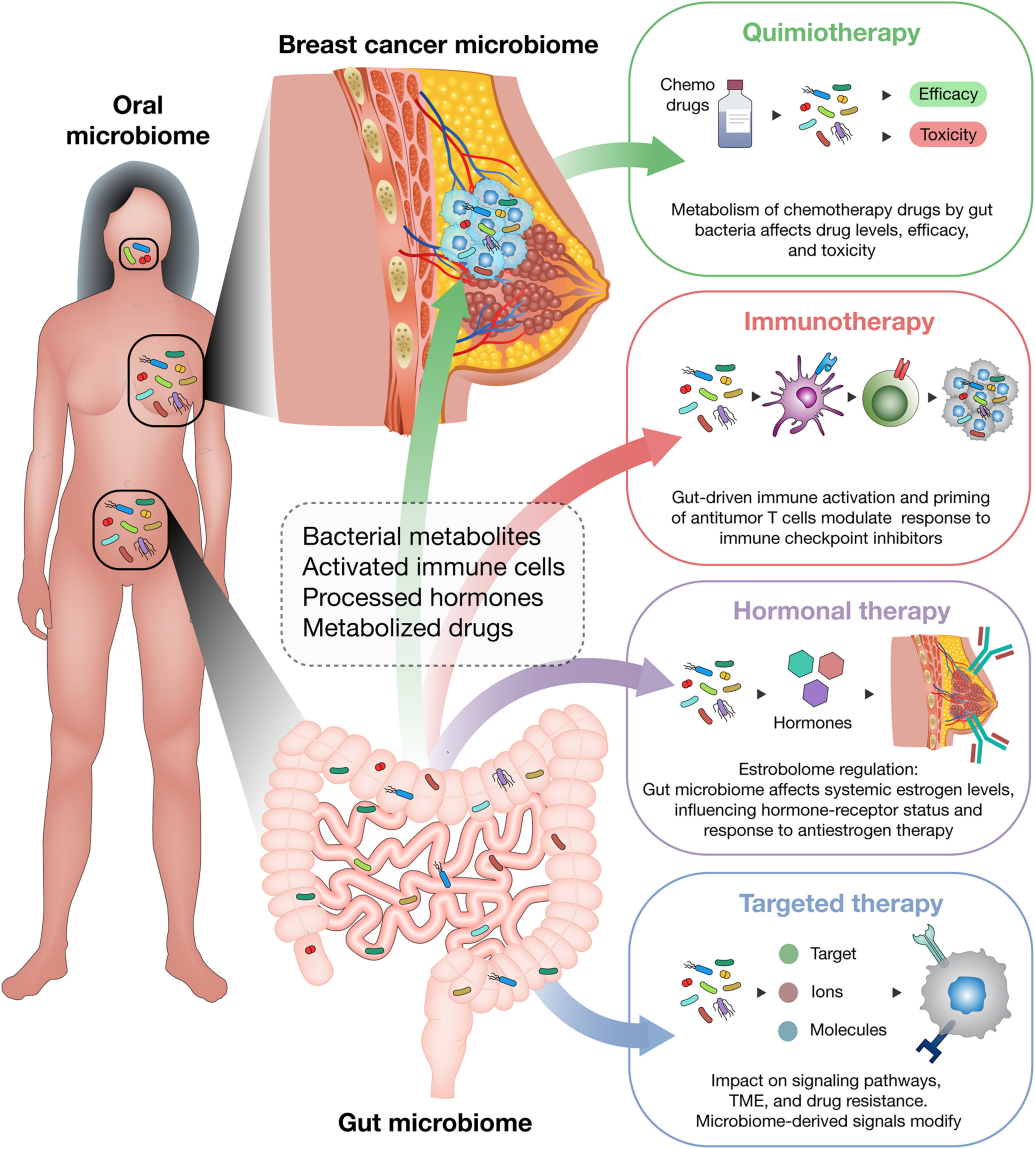
Microbiome–host interactions across breast, gut, and oral compartments shape breast cancer progression and treatment response. This schematic summarizes how microbial ecosystems from the oral cavity, breast tissue, and gut may interact with host physiology to influence breast cancer biology and therapeutic outcomes. The oral microbiome, breast cancer-associated microbiome, and gut microbiome are depicted as interconnected microbial niches that contribute to local and systemic signaling. Within the breast tumor microenvironment, intratumoral and tissue-associated microorganisms may interact with epithelial, stromal, vascular, and immune compartments. The gut microbiome is shown as a major systemic source of bacterial metabolites, activated immune cells, processed hormones, and metabolized drugs, which can reach distant tissues and influence tumor behavior and treatment response. The right panel illustrates four major therapeutic contexts potentially modulated by microbiome-derived signals. In chemotherapy, bacterial metabolism of anticancer agents may alter drug bioavailability, efficacy, and toxicity. In immunotherapy, microbiome-dependent immune priming and systemic immune activation may influence antitumor T-cell responses and sensitivity to immune checkpoint blockade. In hormonal therapy, the estrobolome and other microbiome-associated endocrine functions may affect systemic estrogen levels, hormone-receptor signaling, and responsiveness to antiestrogen treatments. In targeted therapy, microbiome-derived metabolites and inflammatory mediators may influence signaling pathways, the tumor microenvironment, and mechanisms of drug resistance. Together, this Figure highlights the concept that host–microbiome interactions operate across multiple anatomical compartments and may contribute to both breast cancer progression and variation in therapeutic response.

In this context, host–microbiome interactions are examined across breast, gut, and oral environments within a unified analytical framework. Available evidence is interpreted according to its level of validation, distinguishing observations supported in BC from conserved mechanisms described in other malignancies and from findings that remain unconfirmed in mammary systems. This approach enables a controlled interpretation of microbiome-associated processes, delineates the boundaries of current evidence, and highlights areas requiring direct experimental validation. Particular attention is given to direct microbe–host molecular interactions at the cellular interface, which remain comparatively underexplored in BC-focused literature and may have implications for biomarker development and therapeutic targeting (Figure 1).

This narrative review was based on a structured literature search performed in PubMed, Scopus, and Web of Science for articles published up to January 2026. Search terms included combinations of “BC,” “microbiome,” “gut microbiota,” “oral microbiome,” “host–microbe interaction,” “tumor microenvironment,” “therapy response,” “chemotherapy,” and “immunotherapy.” Eligible studies included human observational studies, clinical studies, preclinical models, and mechanistic reports addressing microbiome composition, host–microbiome interactions, therapeutic response, or translational implications in BC. Priority was given to human data, BC–specific evidence, recent publications, and studies with adequate methodological description and appropriate controls. Mechanistic studies from other tumor types were included only when they involved conserved biological pathways relevant to BC and were interpreted as cross-cancer evidence rather than direct BC evidence. Articles were excluded when they lacked methodological clarity, adequate contamination control in low-biomass tissue studies, clinical or mechanistic relevance, or sufficient connection with BC biology. Reference lists of selected articles were also screened to identify additional relevant sources. Evidence was interpreted according to its level of validation, distinguishing observational associations, preclinical mechanisms, clinically supported findings, and extrapolated mechanisms requiring direct confirmation in mammary systems.

## Breast tissue microbiome composition and ecological alterations

Human breast tissue contains a low-biomass microbial community whose composition differs between healthy and malignant states. In healthy breast tissue, dominant bacterial phyla include Proteobacteria, Firmicutes, and Actinobacteria. Reported genera include *Lactobacillus*, *Acetobacter*, and members of the Xanthomonadaceae family (10, 11).

Comparative analyses show differences between healthy and malignant breast tissue. Genera such as *Sphingomonas* and *Pseudomonas* are more frequently detected in non-malignant tissue, whereas *Methylobacterium* is reported at higher abundance in BC cohorts. In luminal-like tumors, increased representation of *Methylobacterium* has been described, indicating variation across tumor subtypes (12, 13).

Breast microbial communities also include non-bacterial microorganisms. Fungal taxa reported in breast tumors include *Trichosporon*, *Geotrichum*, *Epidermophyton*, *Candida*, and *Trichophyton*. Increased abundance of bacterial genera such as *Brevundimonas*, *Actinomyces*, and *Mobiluncus* has also been described in tumor samples. Differences in fungal diversity have been observed between tumor subtypes, with higher diversity reported in luminal-like tumors compared to triple-negative BC (TNBC) in some datasets. Viral sequences from the Polyomaviridae and Hepadnaviridae families, as well as parasitic DNA from *Plasmodium* and *Ascaris*, have been detected in tumor samples (6, 14, 15).

The origin of the breast microbiome remains under investigation. Proposed sources include hematogenous dissemination, migration from neighboring anatomical sites, and colonization through the nipple ductal system. The dense vascular and lymphatic network of breast tissue may facilitate microbial trafficking and transient colonization within this low-biomass environment (16).

Breast tissue represents a distinct microbial niche with compositional variation across physiological conditions, tumor presence, and tumor subtypes. Microbial profiling provides a reference for identifying differences between healthy and malignant states (17, 18).

## Bacterial taxa associated with breast tumorigenesis

Breast tumor tissues frequently display microbial profiles that differ from those observed in adjacent non-malignant tissue. Multiple studies report reproducible shifts in bacterial composition, including enrichment of genera such as *Ralstonia*, *Fusobacterium*, and *Atopobium* in tumor samples compared with surrounding normal tissue. These compositional differences suggest that alterations in microbial community structure represent a reproducible characteristic across tumor samples (10, 19).

High-resolution sequencing combined with culture-based validation has shown that breast tumors generally contain very low quantities of microbial DNA together with restructuring of taxonomic composition. These observations suggest that breast tumor development may be associated with selective ecological disruption rather than simple microbial depletion. Studies incorporating sterile sampling procedures, extensive contamination controls, and matched comparisons between malignant and adjacent tissues have repeatedly reported microbial divergence, supporting the view that these patterns are unlikely to arise solely from technical artifacts (20, 21).

Microbial alterations have also been detected in the ductal system through analyses of nipple aspirate fluid. Because this compartment reflects the ductal-lobular microenvironment, microbial profiles identified in aspirates provide indirect information about ecological conditions along the ductal axis. Individuals with a prior diagnosis of BC often display microbial compositions in nipple aspirates that differ from those observed in healthy individuals, suggesting that microbial shifts may occur within ductal structures during disease development (22, 23).

Comparisons across independent cohorts further indicate that microbial composition differs systematically between non-malignant and cancerous breast tissue. Non-tumor samples often contain bacterial populations that differ from those detected in malignant lesions, whereas tumor samples frequently display enrichment of opportunistic taxa (Table 1) (24, 25). Similar compositional patterns have been reported across multiple sequencing technologies and analytical pipelines, supporting the existence of a reproducible tumor-associated microbial signature rather than cohort-specific observations (26).

**TABLE 1:** Comparative overview of microbiome studies in breast cancer, integrating study design, sample size, evidence strength, and methodological limitations.

References	Biological compartment	Study design	Sample size	Main microbial findings	Evidence strength	Main limitations
Urbaniak et al. (218)	Breast tissue	Human case-control (16S rRNA)	*n* = 43	Differences between tumor and normal tissue; enrichment of *Bacillus* and Enterobacteriaceae	Moderate	Small cohort; low biomass; no functional validation
Urbaniak et al. (219)	Breast tissue	Human observational (16S)	*n* ≈ 80	Breast tissue harbors its own microbiome; not sterile	Moderate	Descriptive; no mechanistic analysis
Hieken et al. (220)	Breast tissue	Human observational (16S)	*n* = 33	Enrichment of *Fusobacterium* and *Atopobium* in tumor tissue	Moderate	Low biomass; small sample size
Hieken et al. (35)	Breast tissue	Human pilot study (multi-omics)	*n* = 33	Microbiome differences linked to stromal and immune features	Moderate	Exploratory; limited cohort
German et al. (13)	Breast tissue	Human observational	*n* ≈ 80	Proteobacteria enrichment in tumor tissue; variability across cohorts	Moderate	Cross-sectional; confounding factors
Kim et al. (221)	Breast tissue	Human observational	*n* ≈ 70	Differences in microbial composition across tumor types	Moderate	No causal inference
Chan et al. (23)	Nipple aspirate fluid	Human case-control	*n* = 48	Differences in ductal microbiome; higher *Alistipes* in BC	Low-Moderate	Indirect sampling; small cohort
Di Modica et al. (172)	Gut microbiome (HER2 + BC)	Human cohort + preclinical	*n* = 24	Microbiome modulates trastuzumab response	Moderate-High	Small cohort; mechanism mainly preclinical
Vernaci et al. (153)	Gut microbiome (TNBC)	Human cohort	*n* = 25	Microbiome correlates with treatment response	Moderate	Exploratory; no validation
Ullern et al. (155)	Gut microbiome (metastatic TNBC)	Human cohort	*n* ≈ 100	Microbial diversity linked to immunotherapy response	Moderate-High	Needs external validation
Gopalakrishnan et al. (44)	Gut microbiome (ICI)	Human + mouse	*n* = 112	Higher diversity improves immunotherapy response	High (cross-cancer)	Not BC-specific
Routy et al. (145)	Gut microbiome (ICI)	Human + FMT	*n* = 249	*Akkermansia muciniphila* associated with better response	High (cross-cancer)	Limited BC data

Differences in microbial composition have also been described across BC molecular subtypes. Luminal-like, HER2-enriched, and triple-negative tumors may display distinct microbial configurations, indicating variability in taxonomic composition across molecular subtypes (25, 27).

Technological developments have recently improved the ability to investigate host-microbe interactions within tumor tissue. Integration of spatial transcriptomics, in situ hybridization, and single-cell metagenomic approaches enables precise localization of microbial cells and characterization of their spatial distribution within tumor architecture. Application of these approaches has begun to clarify the spatial distribution of microbes within breast tumors (28, 29).

Despite these advances, the extremely low microbial biomass of breast tissue continues to present important methodological challenges. Sequencing-based studies remain susceptible to contamination, amplification bias, and batch effects. Robust investigation therefore requires aseptic sampling procedures, extensive negative controls, and complementary validation strategies including quantitative PCR, microscopy, culture-based assays, and shotgun metagenomics. Continued methodological refinement will be essential for clarifying the biological relevance of microbial communities detected in BC tissue (13, 30, 31).

## Commensal bacteria and breast tissue homeostasis

A subset of bacterial taxa detected in non-malignant breast tissue has been associated with ecological features compatible with tissue homeostasis. Genera such as *Lactobacillus*, *Acetobacter*, *Sphingomonas*, and members of the Xanthomonadaceae family have been reported more frequently in healthy or adjacent non-malignant breast samples than in tumor tissue. These findings suggest that non-malignant breast tissue may harbor microbial communities with a more stable ecological configuration, although direct protective effects remain insufficiently demonstrated in human BC (32, 33).

Microbial metabolites represent important mediators of host-microbe communication within breast tissue. Compounds generated through microbial and host co-metabolism, including secondary bile acids, amino-acid derivatives, and short-chain fatty acids (SCFAs), interact with cellular pathways involved in proliferation, immune regulation, and cellular stress responses. Lithocholic acid, produced by certain anaerobic microorganisms, has demonstrated antiproliferative and proapoptotic effects in experimental BC models and has been associated with reduced angiogenic signaling in mammary epithelial cells (34).

The interpretation of commensal bacteria in breast tissue requires caution because most studies describe taxonomic associations rather than functional activity. Differences between non-malignant and malignant tissue may reflect changes in local oxygen tension, stromal composition, vascularization, immune infiltration, or sampling conditions. For this reason, taxa enriched in healthy tissue should not be automatically classified as protective. A more accurate interpretation is that these organisms may represent components of a non-malignant microbial state whose functional relevance remains under investigation (25, 35).

Evidence supporting beneficial roles for breast-associated commensals remains preliminary. Some bacterial groups commonly described in healthy tissues have been linked in experimental or non-breast contexts to epithelial stability, redox balance, or immune equilibrium. However, these effects have not been consistently validated within human breast tissue. Future studies combining spatial localization, culture-based validation, metagenomics, and host transcriptomic profiling will be necessary to determine whether breast-associated commensals actively contribute to tissue homeostasis or merely reflect the ecological conditions of non-malignant tissue (36, 37).

## Gut microbiome and its systemic influence

Alterations in intestinal microbial composition have been consistently reported in individuals with BC, with reproducible differences in community structure compared with healthy controls (17). Sequencing-based studies have identified shifts in the relative abundance of dominant phyla, including Firmicutes, Bacteroidetes, and Proteobacteria, together with compositional changes at the genus level involving taxa such as *Bacteroides*, *Prevotella*, *Clostridium*, and *Ruminococcus*. Several cohorts also describe reduced alpha diversity and increased inter-individual variability, indicating restructuring of microbial community organization rather than uniform depletion (38, 39).

Comparative analyses across independent datasets show that compositional patterns are not fully concordant between studies. Some cohorts report expansion of *Clostridium* clusters, whereas others describe depletion of specific Firmicutes lineages, reflecting variability related to population characteristics and analytical approaches. Despite this heterogeneity, convergent findings indicate that BC is associated with measurable shifts in intestinal microbial composition rather than random variation. Paired analyses using matched controls have confirmed consistent divergence between patient and control profiles, supporting the presence of disease-associated restructuring (40, 41).

Clinical observations indicate that microbial diversity and compositional profiles vary across disease stages and subtypes. Lower diversity and enrichment of Proteobacteria have been more frequently reported in advanced disease, whereas early-stage cases show less pronounced compositional deviation. Differences in relative abundance of genera such as *Bacteroides* and *Prevotella* have also been described across molecular subtypes, suggesting that intestinal microbiome configurations may track with clinical heterogeneity. Longitudinal data indicate that microbial composition may shift during treatment, with partial recovery of diversity in some patients and persistent dysbiosis in others (42, 43).

The intestinal microbiome has also been examined in relation to variability in therapeutic response. Higher microbial diversity has been associated with improved response to immune checkpoint blockade, whereas lower diversity and enrichment of Proteobacteria have been linked to poorer outcomes. Differences in microbial composition have also been associated with variability in chemotherapy tolerance, including increased gastrointestinal toxicity in patients with reduced diversity. Across cohorts, distinct microbial profiles correlate with differential treatment outcomes, supporting an association between intestinal microbiome composition and therapeutic heterogeneity in BC (8, 44, 45).

## Oral microbiome in breast carcinogenesis

Microbial communities from the oral cavity have been examined in relation to BC through their potential systemic dissemination and presence in distant tissues. Several studies have reported the detection of oral-associated bacteria in breast tissue, including *Porphyromonas gingivalis*, *Treponema denticola*, and *Solobacterium moorei*. These findings suggest that microorganisms originating in the oral environment may reach the mammary gland through hematogenous or lymphatic routes. However, the extent to which these bacteria persist or establish stable colonization in breast tissue remains unclear (6, 46, 47).

Periodontal disease has been associated with systemic inflammatory states and altered cytokine profiles. Elevated circulating levels of inflammatory mediators have been reported in individuals with chronic oral infections, suggesting that oral microbial dysbiosis may influence host systemic conditions. Such associations have been described across multiple disease contexts; however, direct evidence linking oral inflammatory profiles with BC development or progression remains limited (48, 49).

Comparative analyses have identified differences in the presence of oral taxa between tumor and adjacent non-malignant breast tissues. Genera commonly associated with periodontal disease and oral dysbiosis have been detected with greater frequency in tumor samples, although results vary across cohorts and methodological approaches. Such observations indicate that oral-derived microorganisms may contribute to the microbial landscape of breast tissue, but do not establish a direct role in tumor initiation or progression.

Among oral microorganisms, *Fusobacterium nucleatum* has received considerable attention because of its detection in multiple solid tumors and its capacity to interact with epithelial and immune cells. Findings from colorectal and melanoma models indicate that *F. nucleatum* promotes epithelial adhesion, inflammatory signaling, and modulation of immune surveillance, its role in BC has not been directly established. Additional oral taxa display diverse immunomodulatory activities. For instance, *Streptococcus salivarius* has been reported to reduce IL-8 secretion and thereby attenuate neutrophil recruitment, whereas *Haemophilus* species have shown inverse associations with circulating C-reactive protein levels, consistent with anti-inflammatory properties. In contrast, *Aggregatibacter* and members of the Pasteurellaceae family can stimulate interferon-γ release from leukocytes, indicating the capacity of oral bacteria to modulate both local and systemic immune responses (50–52).

Hormone-related metabolic interactions provide another possible interface between oral microbial communities and breast tumor biology. Certain bacterial genera, including *Bacteroides* and *Lactobacillus*, produce β-glucuronidase and other enzymes capable of influencing estrogen metabolism and enterohepatic recycling. Modulation of circulating estrogen availability has been proposed as one pathway through which microbial activity could interact with hormone-responsive tissues. Whether oral microbial metabolism contributes meaningfully to these endocrine processes in BC remains to be clarified (53, 54).

Further investigation is required to determine the mechanisms by which oral microorganisms might reach mammary tissue and to define the molecular interactions between microbial profiles, immune signaling, and epithelial homeostasis within the breast microenvironment. Clarifying these processes may also inform preventive strategies targeting oral health, including improved periodontal management and microbiome-modulating interventions, which have been proposed as potential components of broader approaches aimed at reducing cancer-related risk and supporting therapeutic outcomes (55, 56).

## Factors shaping microbiome in breast carcinogenesis

Interindividual variability in microbial composition represents a major source of heterogeneity in BC microbiome research. Reported microbial profiles differ across studies due to variation in host characteristics, environmental exposures, and clinical factors, including age, diet, hormonal status, medication exposure, antibiotic use, and oncologic treatments. Differences in sampling site, disease stage, and treatment history further contribute to variability between cohorts. Microbiome findings should therefore be interpreted in relation to the clinical context of each population rather than as uniform disease-associated patterns (56, 57).

Age is consistently associated with differences in microbial composition. Distinct microbial profiles have been reported between premenopausal, postmenopausal, and older populations. For that reason, age should be considered when comparing microbial data across BC cohorts. Dietary patterns also influence microbial composition, particularly in the intestinal microbiome. Variation in intake of fiber, fats, and refined carbohydrates has been associated with differences in bacterial abundance across individuals. Given the variability in dietary habits between populations, dietary information should be considered when interpreting microbiome data in BC studies (58, 59).

Medication exposure represents another important source of variation. Broad-spectrum antibiotics have been associated with reduced microbial diversity and compositional shifts that may persist after treatment. Information on antibiotic exposure should therefore be included when available. Other medications used in clinical practice, including therapies administered during cancer care, may also coincide with changes in microbial composition. Without adequate clinical annotation, treatment-related variation may be misinterpreted as disease-associated differences (60, 61).

Hormonal status and treatment history further contribute to variability in microbial profiles. Differences have been reported across physiological stages such as reproductive age and menopause, as well as across treatment phases in BC patients. Chemotherapy, endocrine therapy, and radiotherapy have been associated with changes in microbial composition in several studies. For that reason, BC microbiome analyses should account for menopausal status, treatment exposure, and major clinical variables to improve comparability across cohorts (62, 63).

## Mechanisms of microbiome action in breast cancer progression

### Chronic inflammation and tumor microenvironment

Alterations in microbial composition have been associated with changes in host signaling pathways, although direct causal relationships in BC remain incompletely defined, including Toll-like receptors (TLRs) and NOD-like receptors, which detect conserved microbial structures such as lipopolysaccharide, peptidoglycan, and flagellin. Engagement of these receptors initiates intracellular signaling cascades mediated by adaptor molecules such as MYD88, leading to activation of NF-κB and STAT3 transcriptional programs that regulate cytokine production, immune cell recruitment, and stromal remodeling. Persistent activation of these signaling axes has been associated with epithelial proliferation, angiogenesis, and reorganization of tumor-associated stroma, supported by the presence of cytokines including interleukin-1β, interleukin-6, and tumor necrosis factor-α within breast tumor microenvironments. Experimental evidence indicates that epithelial MYD88-dependent signaling enhances tumor progression, while clinical studies report associations between elevated TLR4 expression and adverse outcomes in specific patient cohorts, supporting a mechanistic link between microbial sensing pathways and tumor-associated inflammatory signaling (10, 64).

Spatial and multi-omics profiling studies provide additional insight into how microbial signals are organized within breast tumors. Bacterial DNA and microbial transcripts are not uniformly distributed but appear associated with specific immune and stromal niches. Detection of intratumoral *Fusobacterium* has been linked to transcriptional signatures associated with inflammatory signaling and immune contexture variability, suggesting that microbial presence within tumors may reflect structured host-microbe interactions rather than random contamination (52, 65).

Experimental evidence derived from non-mammary tumor models indicates that *F. nucleatum* can engage TLR4 signaling and promote IL-6–dependent STAT3 activation. These pathways are conserved across tumor types and are also described in BC signaling networks; however, direct functional validation in mammary systems remains limited (66). Mechanistic observations from non-mammary contexts should therefore be interpreted as cross-cancer conserved mechanisms, as shared intermediates such as TLR4, IL-6, and STAT3 are established components of BC biology but have not been specifically linked to *F. nucleatum* activity in breast tissue (67, 68).

Evidence from mammary tumor models also indicates that microbial interventions can modulate inflammatory activity. Administration of selected probiotic strains has been associated with reduced levels of inflammatory cytokines such as IL-6 and TNF-α and partial restoration of cytotoxic immune responses during chemotherapy exposure in murine systems. These effects vary according to bacterial strain, dosage, and host condition, emphasizing the importance of precise microbial characterization when interpreting experimental outcomes (69, 70).

### Direct microbe–host molecular interactions

Physical contact between bacteria and epithelial cells constitutes a mechanism that operates independently of immune-mediated and metabolite-driven signaling. Adhesin–receptor binding, toxin-mediated disruption of junctional proteins, and intracellular bacterial localization enable modulation of host signaling pathways at the cellular interface (71).

*F. nucleatum* expresses the adhesin FadA, which binds E-cadherin and induces β-catenin nuclear translocation. Subsequent activation of β-catenin–dependent transcription drives expression of genes involved in proliferation and invasion. Evidence supporting this mechanism originates from colorectal cancer models and is therefore classified as a conserved cross-cancer process. Comparable FadA-mediated signaling has not been demonstrated in breast tumors (72, 73).

Enterotoxigenic *Bacteroides fragilis* produces the metalloprotease BFT-1, which cleaves E-cadherin and disrupts adherens junctions. Junctional destabilization leads to β-catenin release and activation of NF-κB–dependent transcription. Parallel engagement of Wnt signaling promotes epithelial proliferation and loss of barrier integrity (74, 75). Experimental observations indicate that *B. fragilis* modulates tumor-associated signaling in BC models, although BFT-1 activity has not been directly characterized in human breast tissue (76).

The same organism has also been associated with modulation of tumor cell signaling through a mechanism distinct from classical inflammatory pathways. Experimental breast tumor models report stabilization of NOD1 accompanied by activation of NOTCH signaling. This axis has been linked to expansion of stem-like cellular populations and reduced sensitivity to chemotherapy. Available evidence supports a role in tumor cell plasticity and therapy resistance, whereas direct involvement in inflammatory signaling has not been demonstrated. Interpretation of this pathway therefore aligns with stemness-related programs rather than inflammation (75).

Bacterial internalization provides an additional interaction route. Invasive species penetrate epithelial cells and persist within intracellular compartments, enabling sustained modulation of host signaling (77). Within tumor environments, bacterial communities may organize into biofilm-like structures localized at epithelial surfaces. Such spatial organization has been associated with barrier disruption, inflammatory signaling, and activation of oncogenic pathways, including STAT3. Biofilm-associated architectures have been demonstrated in gastrointestinal tumors but remain unconfirmed in BC (14, 78).

Interactions at the cell–microbe interface have been shown to converge, primarily in preclinical models, on signaling pathways central to BC biology including β-catenin, NF-κB, and epithelial junction regulation. Current evidence supports conservation of these mechanisms across tumor types, whereas functional validation in breast tissue remains limited (79).

### Microbial metabolites as systemic modulators of tumor biology

Microbial metabolism establishes a biochemical interface between intestinal microbial communities and host endocrine and cellular processes through the production of bioactive compounds capable of circulating systemically and interacting with regulatory pathways relevant to tumor biology. One of the most extensively characterized mechanisms involves microbial regulation of estrogen availability through β-glucuronidase activity. After hepatic conjugation, estrogens are excreted into the intestinal lumen as glucuronidated metabolites, where bacterial β-glucuronidases catalyze deconjugation, regenerating biologically active hormones that are subsequently reabsorbed into circulation. This process, collectively defined as the estrobolome, contributes to enterohepatic estrogen recycling and influences systemic hormone exposure in breast tissue, with increased β-glucuronidase activity reported in breast tumor samples, suggesting local regeneration of active estrogens and a direct link between microbial enzymatic function and hormone-dependent signaling pathways relevant to BC biology (54, 80, 81).

Microbial metabolism of dietary compounds provides additional regulatory inputs through the conversion of phytoestrogens into bioactive metabolites such as equol and enterolactone, which are capable of interacting with estrogen receptors and modulating downstream signaling pathways. Observational studies have reported associations between circulating enterolactone concentrations and reduced BC risk, while experimental data indicate effects on receptor signaling and oxidative stress responses in mammary cells, suggesting that microbial transformation of dietary substrates may influence endocrine-related processes involved in tumor development (82–84).

Short-chain fatty acids produced through bacterial fermentation of dietary fiber represent another major class of microbial metabolites with relevance for tumor biology. Butyrate, propionate, and acetate influence gene expression through inhibition of histone deacetylases and activation of G-protein–coupled receptors, leading to modulation of transcriptional programs associated with proliferation, differentiation, and apoptosis. In BC models, butyrate exposure has been associated with reduced cell growth, induction of apoptotic pathways, and decreased epithelial–mesenchymal transition, indicating effects on tumor cell plasticity and invasive potential, although the magnitude of these effects may depend on local concentration, cellular context, and metabolic conditions (85, 86).

Additional microbial metabolites contribute through distinct biochemical pathways. Tryptophan-derived metabolites, including indole derivatives, interact with host transcriptional regulators and have been associated with modulation of cellular differentiation and oxidative stress responses, although direct evidence in BC remains limited. Polyamines such as cadaverine, generated through microbial lysine decarboxylation, have been shown to inhibit migration and invasion in BC models, suggesting a role in regulating epithelial plasticity and metastatic potential (87, 88).

Bile acid metabolism constitutes another microbiome-dependent regulatory axis. Intestinal bacteria convert primary bile acids into secondary derivatives with distinct biological activities. Lithocholic acid has been associated with reduced proliferation and decreased vascular endothelial growth factor expression in BC models through activation of TGR5 signaling, indicating that specific bile acid species may exert tumor-modulating effects depending on their concentration and receptor engagement (89–91).

### Interactions with immune response and immune evasion

Host–microbiome interactions contribute to immune regulation within the breast tumor microenvironment. Immune modulation results from microbial signaling pathways described in the mechanisms section. Microbial-derived signals, particularly those originating from intestinal ecosystems, have been associated with systemic immune variation through continuous interaction with host tissues, influencing cytokine profiles and immune cell recruitment patterns that shape tumor-associated immune infiltrates (10, 92).

Alterations in microbial composition have been associated with shifts in immune cell populations involved in tumor control. Several studies report enrichment of regulatory T lymphocytes and myeloid-derived suppressor cells in contexts of microbial imbalance, accompanied by reduced activity of cytotoxic CD8^+^ T lymphocytes. Macrophage polarization toward tumor-supportive phenotypes has also been described, contributing to immunological tolerance within the tumor microenvironment (93, 94).

Specific microbial taxa have been implicated in immune remodeling across multiple tumor types. *F. nucleatum*, frequently detected in tumor tissues, has been associated with altered antitumor immune responses, including increased recruitment of myeloid populations and reduced cytotoxic lymphocyte activity in experimental models (52, 95). Most available data derive from colorectal cancer, and extrapolation to BC remains limited.

Profiling studies of breast tissue microbiota have identified additional bacterial genera associated with immune and stromal variation within tumors. Genera including *Staphylococcus*, *Clostridium*, and *Adlercreutzia* have been reported in breast specimens and correlate with differences in immune infiltration and extracellular matrix organization. Proteobacterial taxa such as *Sphingomonas* and *Methylobacterium* have also been detected and are associated with variation in immune-related signaling patterns (15, 35, 93).

Microbiome-dependent immune variation has also been linked to therapeutic response. Experimental studies indicate that intestinal microbial composition may influence responsiveness to immune checkpoint blockade, with differences in microbial communities associated with variability in systemic immune activation and T-cell expansion. Transfer of microbiota from responders into germ-free or antibiotic-treated models has been associated with improved antitumor responses, suggesting a role for microbial composition in treatment heterogeneity (96–98).

### Hormonal signaling modification

Estrogen signaling remains a central determinant of BC biology, particularly in hormone receptor-positive subtypes. Microbial metabolism has been proposed as a regulator of systemic estrogen availability through modulation of enterohepatic hormone recycling. Estrogen recycling is mediated by microbial enzymatic activity described in the microbial metabolites section, which influences the circulating pool of bioactive hormones capable of interacting with receptors in peripheral tissues, including the breast (99, 100).

The collection of microbial genes involved in estrogen metabolism has been termed the estrobolome. Multiple intestinal taxa, including members of the genera *Bacteroides*, *Clostridium*, *Escherichia*, and *Bifidobacterium*, have been associated with variability in estrogen-related metabolic capacity. Differences in microbial composition may therefore contribute to interindividual variation in systemic estrogen exposure (101–103).

Changes in microbial diversity have been associated with altered estrogen metabolism across experimental and clinical contexts. Interventions that modify microbial communities, including antibiotics, probiotics, or dietary changes, have been linked to shifts in circulating estrogen metabolite profiles, supporting a dynamic interaction between microbial ecology and endocrine regulation (53, 104).

Population-based studies provide indirect epidemiological support for this relationship. A large case-control investigation involving more than 2,000 women with BC reported a positive association between cumulative antibiotic exposure and subsequent disease risk. Disruption of bacterial populations may alter microbial functions involved in estrogen recycling, potentially modifying systemic hormone exposure (105, 106).

Adiposity represents another factor linking microbiome dynamics and estrogen signaling. In postmenopausal women, adipose tissue becomes the primary site of estrogen synthesis through aromatase-mediated conversion of androgens. Obesity is associated with chronic inflammatory signaling and increased production of cytokines such as IL-6 and TNF-α, which can enhance aromatase expression in adipose and breast stromal tissues. Concurrent alterations in gut microbial composition observed in obesity may further influence microbial functions involved in hormone metabolism (37, 107, 108).

Hormonal changes may also affect microbial communities. Variations in estrogen levels influence immune regulation, epithelial barrier integrity, and bile acid metabolism, factors that shape microbial composition across intestinal and mucosal environments (109). Shifts in microbial diversity have been reported during menopause and in individuals receiving endocrine therapies, indicating bidirectional interactions between endocrine physiology and microbial ecology (110, 111).

Some studies have explored possible functional consequences of tumor-associated microbial alterations. For example, increased β-glucuronidase activity has been reported in breast tumor samples. This enzyme participates in the deconjugation of estrogen metabolites, potentially contributing to local regeneration of biologically active estrogens within breast tissue. Such microbial metabolic activity may represent one mechanism linking microbial profiles with hormone-related signaling pathways relevant to BC biology (112).

Microbial influences may also extend beyond local tissue effects. Tumor-associated bacteria can release extracellular vesicles containing proteins, nucleic acids, and small metabolites capable of interacting with host pathways. These vesicle-mediated signals have been proposed as one mechanism through which localized microbial dysbiosis may interact with systemic processes (113, 114).

Beyond intestinal ecosystems, low-abundance microbial profiles have been detected in breast tissue itself. Organisms such as *Methylobacterium radiotolerans* and *Sphingomonas yanoikuyae* have been identified in mammary specimens. Their functional relevance remains under investigation, although their presence suggests that localized microbial signals may contribute to hormone-related processes within the breast tumor microenvironment (94, 115, 116).

### Subtype-specific contexts in breast cancer microbiome research

Breast cancer molecular subtypes differ in endocrine dependence, immune composition, and therapeutic vulnerabilities. These differences determine the biological context in which microbiome-associated findings should be interpreted and limit the validity of conclusions derived from non-stratified cohorts.

In estrogen receptor-positive (ER+) disease, particularly within luminal A and luminal B subtypes, microbiome-associated variation is most consistently examined in relation to systemic estrogen exposure and endocrine therapy response. Differences in intestinal microbial composition have been associated with variability in circulating estrogen metabolites, suggesting a link between microbial ecology and hormone-dependent signaling (117, 118). Clinical observations further indicate that microbial composition may correlate with endocrine resistance phenotypes and systemic inflammatory profiles in patients receiving aromatase inhibitors or tamoxifen, although available associations remain indirect and influenced by host metabolic status. Within ER+ disease, microbiome-related findings are therefore more appropriately interpreted as modulators of endocrine and metabolic conditions rather than direct determinants of tumor behavior (34, 93).

In HER2-positive BC, microbiome-associated variation has been primarily evaluated in relation to response to HER2-targeted therapy. Cohort analyses indicate that patients with higher baseline gut microbial diversity show improved response to trastuzumab-based regimens, whereas reduced diversity correlates with lower response rates (119). Differences in microbial composition between responders and non-responders suggest that microbial profiles may reflect systemic immune conditions relevant to treatment efficacy. Dependence of HER2-directed antibodies on immune-mediated mechanisms, including antibody-dependent cellular cytotoxicity, provides biological plausibility for such associations. However, available data remain observational, and causal relationships between microbiome composition and treatment response have not been established (120).

Triple-negative breast cancer (TNBC) represents the subtype in which microbiome–immune interactions align most directly with current therapeutic strategies. TNBC is characterized by higher baseline immunogenicity and constitutes the principal BC subtype in which immune checkpoint inhibitors demonstrate clinical benefit. Observational studies in metastatic TNBC cohorts indicate that higher microbial diversity and specific compositional patterns associated with improved outcomes during chemo-immunotherapy, whereas disease progression correlates with microbial configurations enriched in pro-inflammatory taxa (121, 122). Comparable associations have been consistently reported in melanoma and lung cancer, where microbiome composition correlates with variability in response to immune checkpoint blockade. Although such parallels support biological plausibility, direct functional validation in breast-specific systems remains limited (123).

## Impact of the microbiome on therapeutic response in breast cancer

### Chemotherapy

Interactions between intestinal microbial communities and cytotoxic chemotherapy have been associated with variability in therapeutic response, drug efficacy, and treatment-related toxicity. Evidence from microbiological and oncology studies indicates that bacterial activity can modify anticancer agents, potentially influencing drug availability and clinical outcomes. Experimental systems have shown that members of the Enterobacteriaceae family can transform anthracycline compounds under anaerobic conditions. For example, *Raoultella planticola*, *Klebsiella pneumoniae*, and *Escherichia coli* have been reported to alter doxorubicin structure, generating derivatives with reduced cytotoxic activity. Most of these findings originate from preclinical models, and validation in BC patients remains limited (124–126).

Associations between microbiota and chemotherapy-induced immune responses have also been described. Evidence derived primarily from murine tumor models indicates that host–microbiome interactions may contribute to variability in treatment efficacy. In these models, disruption of the intestinal barrier during cyclophosphamide treatment has been linked to systemic immune activation and improved antitumor responses. Reduced treatment efficacy observed in germ-free or antibiotic-treated animals suggests that microbial presence may influence responsiveness to chemotherapy. However, most data derive from non-BC systems, and extrapolation to BC requires caution (127–129).

Cytotoxic therapies can also reshape microbial composition, generating feedback effects associated with treatment tolerability. Paclitaxel exposure has been associated with reduced abundance of commensal taxa and decreased microbial diversity, changes that correlate with increased intestinal toxicity and systemic inflammatory complications in oncology patients. Comparable observations have been reported across multiple cancer types, although data in BC cohorts remain limited (130, 131).

Perturbation of microbial equilibrium may promote intestinal permeability, inflammatory signaling, and systemic complications associated with taxane therapy. Microbiome status has also been linked to variability in response to platinum-based chemotherapy. Studies in animal models show that depletion of commensal microbiota through antibiotic exposure is associated with reduced antitumor efficacy of platinum compounds. Clinical confirmation of this relationship in BC populations remains incomplete, although comparable associations have been observed in other tumor types (132, 133).

Microbial activity has been associated with variability in response to antimetabolite therapies. Experimental findings indicate that bacterial expression of drug-modifying enzymes can either reduce or enhance the activity of nucleoside analogues, depending on the metabolic context. These interactions have been linked to measurable differences in drug sensitivity in vitro, although translation to clinical BC settings remains unclear (134–136).

Observational studies across oncology populations show that microbiome composition correlates with treatment outcomes. Reduced microbial diversity and expansion of opportunistic taxa have been associated with lower response rates and shorter progression-free survival, whereas higher diversity and enrichment of commensal bacteria have been linked to improved therapeutic response. Associations remain preliminary in BC due to limited cohort size and heterogeneity (137–139).

Emerging evidence in BC suggests that specific microbial signatures may be associated with differences in chemotherapy response and disease progression. Multi-omic analyses have identified associations between microbial profiles and treatment resistance. Causality has not been established, and validation in larger, controlled clinical cohorts remains necessary (24, 140, 141).

### Immunotherapy

Immune checkpoint inhibitors (ICIs) restore antitumor immune surveillance by interrupting inhibitory signaling pathways that restrain cytotoxic lymphocyte activity. Therapeutic responses vary considerably among patients, reflecting tumor genomic features, host immune competence, and environmental influences that shape systemic immunity. Interactions between intestinal microbial ecosystems and immune activation have been described, primarily in non-BC clinical cohorts and preclinical models. However, most evidence supporting this interaction originates from melanoma, lung cancer, and renal cell carcinoma cohorts rather than from BC populations (142).

Early experimental work demonstrated that microbial composition can influence immunotherapy outcomes. In murine tumor models, enrichment of *Bifidobacterium* species enhanced dendritic cell maturation and promoted CD8^+^ T-cell priming, resulting in improved tumor control during PD-L1 blockade (143, 144). Clinical observations later reported that patients enriched in *Akkermansia muciniphila* and *Enterococcus hirae* experienced improved responses to PD-1/PD-L1 inhibitors and longer survival, accompanied by enhanced antigen-presenting cell activity and improved epithelial barrier integrity. Evidence linking microbiome composition to immune checkpoint blockade response is strongest in melanoma and lung cancer cohorts, whereas data in BC remain limited and largely associative (145). Functional validation studies further showed that fecal microbiota transplantation from responding individuals restored sensitivity to checkpoint inhibition in germ-free or antibiotic-treated mice, suggesting a potential contribution of microbial communities to immunotherapy efficacy (44). Most of these findings, however, derive from melanoma and lung cancer studies, and direct confirmation in BC remains limited (146).

Microbiome profiling has increasingly been incorporated into clinical immunotherapy research across multiple malignancies. In melanoma and lung cancer cohorts, higher microbial diversity and enrichment of immunostimulatory taxa have been associated with improved therapeutic responses. Bacterial genera such as *Bacteroides*, *Faecalibacterium*, and other SCFAs-producing Firmicutes have been linked to immune activation through enhanced dendritic cell maturation, type I interferon signaling, and cytotoxic lymphocyte recruitment (147, 148). Metagenomic analyses have also reported enrichment of microbial metabolic pathways related to butyrate synthesis, antigen processing, and interferon signaling in patients responding to checkpoint inhibitors (149). Conversely, reduced microbial diversity and expansion of pro-inflammatory taxa have been associated with diminished therapeutic efficacy, potentially through persistent inflammatory signaling and impaired immune activation (150).

Within breast oncology, immune checkpoint blockade currently has established clinical use primarily in TNBC. Treatment outcomes remain heterogeneous, prompting investigation of additional biological determinants that may influence immunotherapy responsiveness. Microbiome analyses in other tumor types indicate that intestinal microbial composition can modulate treatment response; in BC, available data suggest associations between microbial profiles and immune-related features. However, direct evidence linking microbiome composition to immunotherapy outcomes in BC remains limited and requires functional validation (151, 152). Longitudinal microbiome profiling of patients with metastatic TNBC receiving combination regimens that include checkpoint inhibitors has shown that individuals achieving clinical benefit often display higher microbial diversity and enrichment of taxa associated with immune activation following treatment initiation (153). Disease progression, in contrast, has been associated with microbial communities enriched in inflammatory taxa among *Prevotella* species, organisms linked to dysregulated immune signaling and persistent inflammatory responses (154). Current observations therefore suggest a potential contribution of host-microbiome interactions to immunotherapy outcomes in BC, while the strength of evidence remains smaller than that reported for melanoma or lung cancer cohorts (155).

Mechanistic investigations provide biological explanations for these clinical patterns. Commensal microorganisms regulate immune signaling through microbial metabolites, antigenic cross-reactivity, and cytokine network modulation (156). *A. muciniphila* supports epithelial renewal and promotes antigen presentation, whereas *Bifidobacterium* species stimulate type I interferon pathways that facilitate cytotoxic CD8^+^ T-cell expansion. *B. fragilis* contributes to immune equilibrium through polysaccharide-mediated modulation of T-cell differentiation. In contrast, dysbiotic microbial configurations enriched in inflammatory organisms may weaken therapeutic responsiveness. Expansion of taxa such as *Prevotella* spp. or *Clostridium bolteae* has been associated with sustained inflammatory signaling, T-cell exhaustion, and altered recruitment of myeloid populations within the tumor microenvironment (157, 158).

### Hormonal therapy

Endocrine treatment outcomes in hormone receptor-positive BC may be influenced by host-microbiome interactions that shape drug metabolism and systemic hormone regulation. Pharmacomicrobiomics research indicates that intestinal microbial communities may contribute to interindividual variability in drug pharmacokinetics through microbial enzymatic transformation of drug conjugates and modulation of enterohepatic circulation. Tamoxifen, one of the most widely prescribed selective estrogen receptor modulators for reducing BC recurrence, exhibits marked variability in therapeutic response across patients. Experimental and translational studies indicate that bacterial β-glucuronidase enzymes participate in the enterohepatic recycling of tamoxifen metabolites. Hydrolysis of glucuronidated conjugates within the intestinal lumen regenerates pharmacologically active molecules that can be reabsorbed into systemic circulation, thereby altering effective drug exposure and potentially influencing therapeutic efficacy (63, 159).

Microbial metabolism of dietary phytoestrogens may influence endocrine signaling pathways relevant to hormone-responsive malignancies. Several intestinal taxa convert soy-derived isoflavones, among daidzein and genistein, into bioactive derivatives capable of modulating estrogen receptor signaling (160). Certain bacterial populations generate equol from daidzein, a metabolite with selective estrogen receptor modulatory activity that may enhance anti-estrogenic effects in mammary tissue. In contrast, genistein has demonstrated context-dependent estrogenic activity that can interfere with tamoxifen-mediated receptor blockade in experimental systems. Differential microbial processing of dietary isoflavones may contribute to variation in endocrine signaling and could potentially affect responsiveness to hormonal therapy (161, 162).

Aromatase inhibitors may also interact with intestinal microbial ecosystems. Preclinical investigations demonstrate that pharmacological estrogen deprivation can remodel microbial community structure, leading to shifts in taxa associated with host metabolism and inflammatory signaling (163). Murine studies have reported reductions in Bacteroidetes accompanied by expansion of Firmicutes-related groups, including members of the Lachnospiraceae and Ruminococcaceae families, following aromatase inhibitor exposure. Microbial restructuring under endocrine suppression has been associated with increased adiposity, altered lipid metabolism, and heightened inflammatory tone, biological processes that may contribute to metabolic complications and endocrine resistance during long-term therapy (164, 165).

Clinical observations further support a relationship between gut microbial composition and endocrine therapy outcomes in patients with hormone receptor-positive BC. Analyses integrating fecal microbiome profiling with immune and metabolic parameters have identified associations between microbial signatures and treatment resistance. In postmenopausal patients receiving aromatase inhibitors, enrichment of taxa belonging to the Veillonellaceae family has been linked to endocrine-resistant phenotypes. Members of this microbial group participate in metabolic pathways that influence host energy balance and systemic inflammatory signaling. Correlations between microbiome composition, circulating cytokines, and tumor-infiltrating lymphocyte density suggest that microbial-driven inflammatory environments may contribute to immune suppression during endocrine therapy (166, 167).

Microbiome composition has also been explored as a potential predictor of treatment response in advanced disease treated with endocrine-based targeted regimens. Studies evaluating patients with metastatic hormone receptor-positive BC receiving CDK4/6 inhibitors combined with endocrine therapy have reported associations between fecal microbial signatures and systemic immune markers, notably the neutrophil-to-lymphocyte ratio. Lower baseline inflammatory indices correlate with improved therapeutic responses and longer progression-free survival. Conversely, enrichment of taxa such as *Clostridium innocuum*, *Oscillibacter*, and *Eubacterium hallii* has been linked to elevated inflammatory profiles and inferior clinical outcomes. Microbial modulation of host inflammatory tone may therefore represent one mechanism influencing sensitivity to endocrine-targeted treatments in advanced BC (168–170).

### Targeted therapy

Amplification or overexpression of HER2 occurs in approximately one fifth of BC and defines a molecular subtype characterized by aggressive clinical behavior but high susceptibility to targeted therapy. Monoclonal antibodies and antibody-drug conjugates directed against HER2 have substantially improved survival outcomes in affected patients. Therapeutic agents including trastuzumab, pertuzumab, trastuzumab emtansine (T-DM1), and trastuzumab deruxtecan (T-DXd) exert antitumor activity through complementary mechanisms involving blockade of HER2-driven signaling pathways, receptor internalization, and immune-mediated cytotoxicity. Pertuzumab prevents HER2/HER3 heterodimerization and thereby suppresses activation of downstream PI3K-AKT signaling, whereas T-DM1 and T-DXd function as antibody-drug conjugates delivering cytotoxic payloads selectively to HER2-overexpressing tumor cells. Long-term clinical follow-up from the CLEOPATRA trial demonstrated that combining trastuzumab and pertuzumab with chemotherapy significantly improves overall survival in HER2-positive metastatic BC, establishing dual HER2 blockade as a central therapeutic strategy (171).

Clinical observations suggest that intestinal microbial ecosystems may contribute to variability in therapeutic response to HER2-directed treatment. Analysis of patients with HER2-positive BC receiving neoadjuvant trastuzumab-based regimens revealed that responders exhibited higher baseline gut microbiome α-diversity than non-responders. Favorable responses were associated with enrichment of microbial taxa belonging to Clostridiales, Bifidobacteriaceae, Turicibacteriaceae, and Prevotellaceae, organisms commonly linked to immune homeostasis and anti-inflammatory signaling. In contrast, non-responders showed increased relative abundance of Bacteroides species, microbial groups associated with pro-inflammatory metabolic profiles and altered immune regulation. Differences in microbial diversity and taxonomic composition therefore appear to shape systemic immune conditions that support trastuzumab-mediated antitumor activity (172).

Interactions between intestinal microbial communities and host immunity may provide a mechanistic explanation for variability in treatment response. Microbial metabolites produced in the gut have been reported to influence dendritic cell maturation, cytokine secretion, and cytotoxic lymphocyte activation in several experimental systems. Immunological modulation driven by microbial metabolism can strengthen antibody-dependent cellular cytotoxicity and adaptive immune responses that support HER2-directed therapy. Conversely, dysbiotic microbial configurations may weaken immune surveillance through impaired antigen presentation and reduced expression of cytolytic mediators, perforin and granzyme B. Crosstalk linking intestinal microbiota with systemic immune regulation therefore represents a biological axis capable of influencing therapeutic efficacy during HER2-targeted treatment (8, 66).

Microbial influences may extend beyond HER2-directed agents to additional targeted therapies used in BC management. Cyclin-dependent kinase 4/6 inhibitors, palbociclib, ribociclib, and abemaciclib, are commonly administered in hormone receptor-positive disease to restrain cell-cycle progression through inhibition of the G1/S transition. Preliminary clinical observations suggest that baseline gut microbiome composition and systemic inflammatory profiles may correlate with therapeutic outcomes among patients receiving CDK4/6 inhibitors in combination with endocrine therapy, although mechanistic confirmation remains limited. Interactions between host physiology and bacterial populations affecting immune regulation, metabolic signaling, and inflammatory balance have therefore been proposed as possible determinants of treatment response. Similar regulatory frameworks have also been proposed for poly(ADP-ribose) polymerase inhibitors, particularly olaparib and talazoparib. Both agents exploit synthetic lethality in tumors harboring *BRCA1* or *BRCA2* mutations, and microbiota-related modulation of oxidative stress pathways has been proposed as a possible factor influencing therapeutic activity, although direct evidence remains limited (173).

## Microbiome modulation as a therapeutic strategy in breast cancer

### Dietary and lifestyle modulation of the microbiome

Dietary patterns and lifestyle behaviors represent major modifiable determinants of gut microbiome composition, with direct effects on microbial diversity and community structure. Nutritional intake can rapidly influence microbial profiles, promoting shifts in taxonomic composition within short timeframes. Diets enriched in plant-derived foods, complex carbohydrates, and unsaturated fatty acids, characteristic of Mediterranean-style dietary patterns, have been consistently associated with increased microbial diversity and enrichment of commensal taxa including *A. muciniphila*, *Faecalibacterium prausnitzii*, and *Roseburia* species (174–176).

In contrast, dietary patterns characterized by high intake of refined carbohydrates, saturated fats, and low fiber consumption are associated with reduced microbial diversity and compositional shifts toward taxa frequently reported in metabolically unfavorable profiles. Limited intake of fermentable substrates constrains the expansion of beneficial commensal populations, favoring ecological imbalance within the intestinal microbiome (177).

Nutrient composition imposes selective ecological pressure on intestinal microbial communities. Prebiotic substrates such as inulin and fructooligosaccharides promote the expansion of genera including *Bifidobacterium* and *Lactobacillus*, contributing to increased microbial richness. Plant-derived bioactive compounds, particularly polyphenols and unsaturated fatty acids, further influence microbial composition by acting as substrates for specific commensal groups. Conversely, diets enriched in animal-derived saturated fats and low in fiber are associated with enrichment of genera such as *Bacteroides* and *Alistipes*, reflecting shifts in community structure linked to dietary patterns (178, 179).

Physical activity interacts with dietary exposures to influence gut microbial ecology. Observational and interventional studies report associations between regular exercise and increased microbial diversity, along with enrichment of specific bacterial taxa (180). Exercise has also been associated with stabilization of microbial community structure across individuals, suggesting a role in maintaining ecological balance (181).

Additional lifestyle determinants contribute to shaping microbial composition. Sleep restriction and circadian disruption have been associated with alterations in microbial rhythmicity and reduced diversity (182). Chronic alcohol consumption is linked to compositional shifts characterized by reduction of commensal taxa and expansion of opportunistic microorganisms, whereas partial recovery of microbial profiles has been observed following sustained abstinence. Environmental context further influences microbial diversity, with individuals in urban settings typically showing lower microbial richness compared to rural populations, reflecting differences in environmental exposure and lifestyle patterns (183, 184).

### Use of probiotics and prebiotics

Microbiome-directed interventions have been explored as potential adjunct approaches in BC prevention and management. Although probiotic and prebiotic supplementation has not yet been incorporated into standard oncological guidelines, modulation of intestinal microbial ecosystems may influence host metabolic, endocrine, and immune pathways that participate in breast tumor development. One important mechanism involves microbial regulation of estrogen metabolism through the estrobolome, a functional group of bacterial enzymes capable of deconjugating estrogens and modifying systemic hormone availability. Additional pathways include attenuation of inflammatory signaling cascades, regulation of oxidative stress, and modulation of immune surveillance, all of which contribute to the biological landscape that shapes mammary carcinogenesis (185, 186).

Experimental studies provide mechanistic evidence suggesting potential antitumor effects of selected probiotic strains. Investigations employing *Lactobacillus rhamnosus*, *Lactobacillus casei*, and *Bifidobacterium longum* in BC cell systems and murine tumor models have been associated with reduced tumor burden and changes in apoptotic signaling in preclinical models, and attenuation of metastatic dissemination. Immune profiling in these models indicates activation of cytotoxic lymphocyte responses, reduced production of pro-tumorigenic inflammatory mediators, and modulation of angiogenic signaling pathways within the tumor microenvironment. Microbial metabolites and secondary bile acids appear to mediate part of this immune remodeling, linking microbial metabolic activity with tumor-associated immune regulation. Evidence supporting probiotic use in breast cancer remains primarily preclinical, and clinical validation is limited by small cohorts and heterogeneous study designs (187–189).

Microbiome modulation may also influence treatment tolerance and therapeutic responses during systemic cancer therapy. Several clinical and translational studies suggest that probiotic supplementation can mitigate chemotherapy-associated dysbiosis, reinforce intestinal epithelial barrier integrity, and reduce gastrointestinal toxicity during treatment. Preservation of microbial homeostasis may stabilize host immune and metabolic responses under cytotoxic stress, potentially improving treatment tolerance and overall patient resilience. Nevertheless, substantial heterogeneity across clinical trials, including variation in probiotic strains, dosing strategies, and patient populations, continues to limit definitive conclusions regarding therapeutic efficacy (188, 190).

Epidemiological observations further support a possible preventive role for probiotic-rich dietary exposures. Large population-based analyses have reported inverse associations between intake of fermented dairy products containing *L. casei* Shirota and BC incidence (191). Microbiome-mediated modulation of estrogen recycling, regulation of systemic inflammatory tone, and improvements in metabolic homeostasis represent plausible biological mechanisms underlying these associations. Although observational findings cannot establish causality, endocrine-microbial interactions provide a mechanistic framework linking microbial ecology with BC susceptibility (192, 193).

### Fecal microbiota transplantation as an emerging approach

Fecal microbiota transplantation (FMT), initially developed to treat recurrent *Clostridioides difficile* infection, has been investigated in experimental and early-phase clinical settings as a strategy to restore intestinal microbial homeostasis and modulate host immune function (194). Transfer of complex microbial communities from healthy donors can reconstitute microbial diversity, reinforce epithelial barrier integrity, and influence systemic inflammatory signaling pathways involved in antitumor immunity. Reestablishment of balanced microbial ecosystems has been proposed as a means to modify immune competence during cancer therapy. Experimental and translational studies indicate that restructuring gut microbial profiles through FMT can influence systemic immune activation and tumor-immune interactions in patients receiving immune-modulating treatments (195–197).

Clinical investigations have begun to evaluate the therapeutic implications of microbiome transfer in patients with advanced malignancies. A randomized phase Ib/IIa clinical protocol has been designed to determine whether fecal microbiota transplantation derived from immune checkpoint inhibitor responders can restore sensitivity to anti-PD-1 therapy in patients with refractory metastatic melanoma. The study integrates longitudinal microbiome profiling, metabolomic analyses, and immune phenotyping to examine whether donor microbial composition influences treatment outcomes and antitumor immune activation (198). Complementary translational observations have further supported the therapeutic relevance of microbiome reconstitution in oncology. Recent analyses indicate that fecal microbiota transfer from immunotherapy-responsive donors can reprogram microbial ecosystems and promote immune reactivation in treatment-resistant solid tumors, suggesting that microbiome-directed interventions may contribute to resensitizing tumors to immune checkpoint blockade across multiple malignancies (199). Microbiome profiling demonstrated enrichment of taxa associated with favorable immunotherapy outcomes, supporting the view that donor microbial composition influences systemic immune activation and therapeutic response (200).

Integrated analyses of clinical cohorts further highlight the importance of microbial composition in determining FMT efficacy. Microbiota derived from immunotherapy responders have consistently produced stronger therapeutic benefits than microbiota obtained from non-responders. Across multiple studies, enrichment of bacterial taxa such as *A. muciniphila*, *F. prausnitzii*, and *Bifidobacterium* species has been associated with improved immune activation and better clinical outcomes following immune checkpoint blockade (195). Capsule-based FMT have also demonstrated improved preservation of microbial diversity compared with colonoscopic infusion, suggesting a minimally invasive route for microbiome restoration (201). Most clinical evidence derives from melanoma and other solid tumors, and direct evidence in breast cancer is currently lacking.

## Limitations and challenges in translating microbiome-therapy interactions in breast cancer

Research examining the interaction between microbial profiles and BC therapies has expanded rapidly in recent years; however, several methodological and conceptual limitations continue to restrict translation into clinical oncology. One of the most persistent technical challenges arises from the extremely low microbial biomass detected in mammary tissue and tumor samples. In these settings, microbial DNA concentrations frequently approach the lower detection limits of sequencing technologies, making datasets highly susceptible to contamination introduced during sample processing, reagent preparation, or sequencing workflows. Studies evaluating low-biomass microbiomes demonstrate that background microbial DNA originating from laboratory reagents can generate misleading taxonomic profiles when negative controls and decontamination strategies are not rigorously implemented. Because many breast tumor microbiome investigations rely on low microbial biomass and trace bacterial signals, discrimination between authentic tumor-associated taxa and environmental contaminants remains challenging without strict experimental controls and standardized reporting (202). Such technical artifacts complicate interpretation of intratumoral microbiome data and limit reproducibility across independent cohorts. In parallel, a substantial proportion of mechanistic insight in microbiome–oncology research derives from non-BC models; although several signaling pathways are conserved, relevance in BC requires direct experimental validation and cannot be inferred solely from cross-tumor evidence (203).

Low microbial biomass not only increases susceptibility to contamination but also affects reproducibility across studies. Background DNA derived from extraction kits, PCR reagents, and laboratory environments can dominate sequencing signals when true microbial load is minimal, leading to potential misclassification of common contaminant taxa such as *Ralstonia*, *Sphingomonas*, and *Pseudomonas*, which are also frequently reported in breast tissue datasets. Inconsistent implementation of negative controls, variability in sample processing, sequencing depth, and bioinformatic filtering further contribute to inter-study discrepancies, limiting cross-cohort validation of microbial signatures. Rigorous experimental design, including systematic use of blank controls, quantitative assessment of microbial load, and orthogonal validation methods such as qPCR or in situ hybridization, is therefore required to distinguish true intratumoral microbial signals from technical artifacts (204, 205).

Interpretation is further complicated by substantial methodological heterogeneity across microbiome studies in breast oncology. Investigations frequently examine different biological compartments, ranging from fecal samples and circulating microbial DNA to normal breast tissue and tumor biopsies, each representing distinct ecological niches and therefore distinct microbial compositions. Analytical strategies also vary widely, ranging from 16S rRNA amplicon sequencing to whole-metagenome approaches, while bioinformatic pipelines for taxonomic classification and contamination filtering differ substantially between laboratories. Such variability makes direct comparison across studies difficult and limits the reproducibility of microbial signatures associated with therapeutic response. Clinical characteristics of the host introduce an additional layer of complexity. Factors such as menopausal status, obesity, diet, metabolic disorders, and antibiotic exposure can independently shape microbial communities, creating potential confounding effects when evaluating associations between microbiota and cancer biology. Analyses of breast tissue microbiota have shown that several host-related variables correlate with microbial composition, indicating that observed microbial differences may reflect patient characteristics rather than tumor-specific processes (13, 79).

Another limitation concerns the currently modest predictive value of microbiome-based biomarkers for therapeutic outcomes. Although several clinical studies have explored whether baseline microbial composition can forecast response to chemotherapy or immunotherapy, results remain inconsistent and frequently lack external validation. A prospective multicenter cohort evaluating the gut microbiota of patients receiving neoadjuvant chemotherapy identified microbial patterns associated with treatment response, yet the predictive capacity of these signatures varied considerably across individuals and required confirmation in independent populations (206). Similar findings have emerged in metastatic TNBC, where higher gut microbial diversity has been associated with improved outcomes during chemo-immunotherapy regimens. Despite these encouraging observations, available datasets remain relatively small, and predictive models have not yet achieved sufficient accuracy or reproducibility to support clinical implementation (155).

Mechanistic studies have begun to clarify how microbial metabolism may influence pharmacological responses, yet translating these discoveries into actionable biomarkers remains challenging. Experimental pharmacomicrobiomics research has shown that variability in gut microbial composition can affect the metabolism of endocrine therapies such as tamoxifen, potentially modifying systemic exposure to active metabolites through microbial enzymatic activity and enterohepatic recycling pathways (207). Nevertheless, observational analyses in patients undergoing tamoxifen therapy have not consistently demonstrated robust associations between microbial composition and circulating endoxifen levels once statistical correction for multiple comparisons is applied (63).

A related concern involves the predominance of correlative observations rather than direct demonstration of causality. Many reported associations between microbial taxa and therapeutic outcomes arise from sequencing-based profiling studies that identify statistical relationships without establishing functional mechanisms. Although experimental models indicate that microbial metabolites can influence immune signaling, inflammation, and hormone metabolism, direct verification of these processes within human breast tumors is still scarce. Reviews addressing microbiome-therapy interactions emphasize that most available evidence relies on observational data, whereas integrative approaches combining metagenomics with metabolomic, transcriptomic, and functional analyses remain comparatively limited. Without experimental confirmation of microbial activity within host tissues, it remains difficult to determine whether microbial signatures actively shape therapeutic response or simply reflect physiological changes induced by treatment (8, 208, 209).

A structured separation between observational, preclinical, and clinical evidence is therefore required when interpreting microbiome findings in BC. Observational data identify correlations between microbial profiles and clinical or molecular features, whereas preclinical models provide mechanistic plausibility under controlled conditions. Clinical studies provide translational relevance, but many remain limited by small cohorts, heterogeneous treatments, and insufficient longitudinal validation, as highlighted in microbiome–oncology research frameworks (79, 210).

Finally, a substantial gap persists between mechanistic discoveries and clinical application. Preclinical studies increasingly demonstrate that microbial ecosystems can influence chemotherapy metabolism, endocrine signaling pathways, and antitumor immune responses, suggesting potential opportunities for microbiome-directed therapeutic strategies. Most proposed microbiome-targeted interventions, comprising dietary modification, probiotic supplementation, and microbiota transplantation, are still limited to preclinical systems or early exploratory clinical studies. Analyses of the intratumoral microbiome have suggested that bacterial communities within breast tumors may be associated with response to chemo-immunotherapy in TNBC. However, confirmation in larger prospective cohorts remains necessary before such observations can be translated into clinically actionable strategies (211). Consequently, while microbiome research has revealed new biological dimensions of therapy response, substantial methodological refinement, rigorous functional validation, and well-designed clinical trials will be necessary before microbiome-informed strategies can be incorporated into routine BC management (79, 203).

A recurring issue in the current literature is the tendency to combine heterogeneous evidence without separating findings generated in breast cancer from mechanisms described in other tumor types, leading to potential overinterpretation of biological relevance. Claims regarding microbiome-based interventions, including probiotics, fecal microbiota transplantation, and microbiome-derived biomarkers, should therefore be interpreted with caution in breast cancer, as most available evidence remains preclinical or derived from other malignancies. Breast cancer–specific data are limited and largely associative, and clinical applicability has not yet been established, requiring prospective validation in well-controlled studies.

## Conclusion and future perspectives

Breast cancer develops within a complex biological context shaped by endocrine, immune, metabolic, and environmental influences. Within this framework, microbial communities present in the gut, oral cavity, and mammary tissue have been linked to processes that influence inflammation, immune regulation, and hormone metabolism. Differences in microbial composition between healthy and malignant breast tissue, together with systemic effects of gut-derived metabolites, indicate that host-microbiome interactions have been associated with variability in tumor biology and treatment response, although causality remains to be established. At present, however, most observations describe ecological associations rather than direct microbial activity within breast tumors (10, 93).

Several biological pathways provide plausible connections between microbial ecosystems and BC progression. Microbial metabolites, including SCFAs, bile acid derivatives, and tryptophan-related compounds, influence immune signaling, epithelial differentiation, and chromatin regulation. Hormone metabolism also represents a potential interface, as bacterial β-glucuronidase activity can modify enterohepatic estrogen circulation, a process relevant to hormone receptor-positive disease (54, 81). Although such mechanisms are biologically consistent with tumor-related pathways, the extent to which microbial processes actively shape human breast tumor development remains incompletely defined.

Interpretation of current findings is limited by methodological variability across studies. Breast tissue represents a low-biomass microbial environment, making sequencing results vulnerable to contamination and technical artifacts. Differences in sampling strategies, sequencing platforms, contamination filtering, and taxonomic classification pipelines further complicate comparisons across cohorts. In addition, host-related determinants, including age, diet, antibiotic exposure, metabolic status, and menopausal transition, substantially influence microbial composition, introducing additional layers of heterogeneity that must be carefully controlled in future research (13, 20, 212).

Addressing these limitations will require well-designed longitudinal and multicenter investigations supported by standardized analytical frameworks. Greater emphasis on microbial function rather than taxonomic presence alone will also be important. Integrative approaches combining metagenomics, metabolomics, spatial transcriptomics, and immune profiling may clarify how microbial signals interact with host cellular programs within tumor microenvironments (213–215). Combined profiling approaches that incorporate metagenomic, metabolomic, transcriptomic, and immunophenotypic data are therefore expected to reveal pathway-level microbial signatures with stronger biological and clinical relevance than associations based solely on individual taxa (141).

From a clinical perspective, microbiome-informed strategies remain exploratory. Microbial signatures could eventually complement molecular and immunological biomarkers used to stratify patients, particularly if reproducible functional pathways are identified. Interventions aimed at modifying microbial ecosystems, including dietary modulation, microbial supplementation, or microbiota transplantation, are currently under investigation but require carefully controlled trials before integration into routine oncology practice (195, 216, 217).

Integration of microbial signals across breast, gut, and oral compartments provides a more coherent view of host–microbiome interactions in BC. Interpreting findings according to their level of experimental support allows separation of mechanisms grounded in BC from those that remain indirect. Such distinctions are likely to influence study design and may support the identification of microbiome-associated biomarkers and therapeutic strategies.

A clearer understanding of host-microbiome interactions in BC will depend on studies capable of linking microbial ecology with functional activity and clinical outcomes. Such evidence will determine whether microbial features represent indirect markers of host physiology or components of tumor-associated biological processes with translational relevance.
